# Simultaneous localization and mapping in a multi-robot system in a dynamic environment with unknown initial correspondence

**DOI:** 10.3389/frobt.2023.1291672

**Published:** 2024-01-11

**Authors:** Hadiseh Malakouti-Khah, Nargess Sadeghzadeh-Nokhodberiz, Allahyar Montazeri

**Affiliations:** ^1^ Department of Control Engineering, Qom University of Technology, Qom, Iran; ^2^ School of Engineering, Lancaster University, Lancaster, United Kingdom

**Keywords:** SLAM, multi-robot SLAM, Fast-SLAM, dynamic environments, moving landmarks, map merging

## Abstract

A basic assumption in most approaches to simultaneous localization and mapping (SLAM) is the static nature of the environment. In recent years, some research has been devoted to the field of SLAM in dynamic environments. However, most of the studies conducted in this field have implemented SLAM by removing and filtering the moving landmarks. Moreover, the use of several robots in large, complex, and dynamic environments can significantly improve performance on the localization and mapping task, which has attracted many researchers to this problem more recently. In multi-robot SLAM, the robots can cooperate in a decentralized manner without the need for a central processing center to obtain their positions and a more precise map of the environment. In this article, a new decentralized approach is presented for multi-robot SLAM problems in dynamic environments with unknown initial correspondence. The proposed method applies a modified Fast-SLAM method, which implements SLAM in a decentralized manner by considering moving landmarks in the environment. Due to the unknown initial correspondence of the robots, a geographical approach is embedded in the proposed algorithm to align and merge their maps. Data association is also embedded in the algorithm; this is performed using the measurement predictions in the SLAM process of each robot. Finally, simulation results are provided to demonstrate the performance of the proposed method.

## 1 Introduction

Simultaneous localization and mapping (SLAM) is one of the most important developments in the field of robotics, enabling robots operating in GPS-denied environments to perceive their surroundings and localize themselves within the identified map. This is especially useful for industries such as underground mining and nuclear decommissioning, where mobile robots are required to explore and complete various tasks, such as maintenance, inspection, and transportation, in environments that are inaccessible and hazardous for humans. These missions are accomplished either using a single robot in isolation (Montazeri et al., 2021) or using a team of cooperative robots (Burrell et al., 2018). The robot or robots need to cope with the time-varying, restricted, uncertain, and unstructured nature of the environment to achieve planning and execution of the necessary tasks. This in turn requires the design and development of advanced motion control and navigation algorithms, along with strong cognitive capabilities in order for the robot to perceive the surrounding environment effectively. The use of both single- and multi-robot platforms can be advantageous, depending on the specific application and environment.

SLAM-based navigation refers to the techniques by which the robots simultaneously localize themselves in an unknown environment where a map is required. This technique can be applied to single-robot (Debeunne and Vivet, 2020) or multi-robot (Almadhoun et al., 2019) systems, as well as in either static (Kretzschmar et al., 2010) or dynamic (Saputra et al., 2018) environments.

In a static environment, landmarks are fixed, which means that all objects in the environment are stationary and none are moving (Stachniss et al., 2016). A dynamic environment is an environment in which objects are moving, such as an environment that includes humans, robots, and other means of transportation. In a dynamic environment, the SLAM process becomes more complex due to continuous changes in the environment (such as moving objects, changing colors, and combinations of shadows), and this makes the problem more challenging, for example, by creating a mismatch between previous and new data (Li et al., 2021).

Several successful solutions have been presented for the single-robot and multi-robot SLAM problem in static environments (Saeedi et al., 2016; Lluvia et al., 2021). However, the SLAM problem in a dynamic environment is more complicated due to the requirements for simultaneous detection, classification, and tracking of moving landmarks. In recent years, single-robot and multi-robot SLAM problems in dynamic environments have attracted the attention of many researchers. In (Vidal et al., 2015; Badalkhani and Havangi, 2021), solutions for solving the single-robot and multi-robot SLAM problems in dynamic environments are presented. In (Vidal et al., 2015), a method is presented for SLAM in which only static (fixed) landmarks are considered and moving ones are omitted from the map. The paper uses an outlier filter and separates fixed and moving landmarks, which are included in a set of negligible signs; the authors only use fixed landmarks to implement their solution to the SLAM problem and do not consider moving landmarks.

In (Badalkhani and Havangi, 2021), a solution for the multi-robot SLAM problem in a dynamic environment is presented. The proposed SLAM-based method is developed using a Kalman filter (KF); it detects moving landmarks based on possible location constraints and expected landmark area, which enables detection and filtering of moving landmarks. The main goal of (Badalkhani and Havangi, 2021) is the development of a new method to identify the moving parts of the environment and remove them from the map.

There are also some pieces of research implementing the SLAM problem in a dynamic environment through object recognition or using image processing techniques. However, some of these methods, such as those presented in (Liu et al., 2019; Pancham et al., 2020; Chen et al., 2021; Theodorou et al., 2022; Guan et al., 2023), also aim to remove moving landmarks found in the dynamic environment.

As mentioned, researchers who have investigated the problem of SLAM in a dynamic environment using one or more robots have mostly attempted to implement SLAM through the elimination of the moving landmarks, with only fixed ones being retained for the mapping procedure, which leads to the failure of the method when the environment is complex, with many moving objects, or in the absence of fixed objects.

In this paper, in contrast with previous work, a decentralized multi-robot modified Fast-SLAM-based algorithm is designed for use in a dynamic environment where all the landmarks are moving. It is worth mentioning that, in Fast-SLAM (Montemerlo et al., 2002), particle filtering (PF) (Godsill, 2019) is used to handle the non-linear kinematics of the robots via the Monte Carlo technique. Fast-SLAM is a filtering-based approach to SLAM decomposed into a robot localization problem, and a collection of landmark estimation problems conditioned on the robot pose estimate. This can be obtained using Bayes’ rule combined with the statistical independence of landmark positions which leads to Rao Blackwellized particle filtering (RBPF) SLAM (Sadeghzadeh-Nokhodberiz et al., 2021).

In this study, we have simplified the multi-robot Fast-SLAM problem by considering two wheel-based robots in a dynamic environment with two moving landmarks with a known kinematic model. The proposed method can easily be extended to cases with more robots and landmarks. Each robot searches the environment and observes it with its onboard lidar sensor. Here, it is assumed that the kinematic models of the robots and landmarks are known, which makes the use of a modified Fast-SLAM method meaningful. This method is based on observer use of a particle filter and extended Kalman filter (EKF). However, several modifications of the normal Fast-SLAM method are required. The first modification is adding a prediction step to the EKF used for mapping using the kinematic model of the landmarks. Adding this step to the algorithm does not obviate the need for initialization, as the initial values of the landmarks’ positions are assumed to be unknown. This prediction step is performed using the landmark’s kinematic model at every sample time after the first visit to the landmark, even if the landmark has not been visited again by the lidar sensor. The second modification is that, in contrast with the normal approaches to data association with static landmarks, data association is performed based on the predicted measurement obtained from the predicted map. When data association is performed based on the predicted position of a landmark, it is less likely that a previously visited landmark will be wrongly diagnosed as a new one due to its movement. As the third modification, and due to the unknown initial correspondence, coordinate alignment and map-merging are added to the algorithm. In this step, the relative transformation matrix of the robots’ inertial frames is computed when the robots meet each other, using a geographical approach as presented in (Zhou and Roumeliotis, 2006; Romero and Costa, 2010) to compute this transformation matrix, and the maps obtained in the coordinate system of each robot are then fused and merged.

In summary, the novelty of this paper can be considered to be the extension of the Fast-SLAM algorithm in several aspects, as follows:

1. Addition of a prediction step to the EKF used for mapping, based on the kinematic model of the landmarks after the first visit to each landmark and at every sample time.
2. Data association based on the predicted measurements obtained from the predicted map.
3. Addition of coordinate alignment and map-merging to the algorithm when the robots meet each other.

The paper is organized as follows. In Section 2, a system overview is presented, including a kinematic model of the robots and landmarks as well as the lidar measurement model. The modified Fast-SLAM algorithm for the dynamic environment is proposed in Section 3. Section 4 describes the coordinate alignment and map-merging problem. Simulation results are presented in Section 5, and conclusions are provided in Section 6.

## 2 System overview

In this paper, for the sake of simplicity, two mobile robots and two dynamic landmarks have been considered. As an example, differential drive mobile robots such as the Pioneer P3-DX (Zaman et al., 2011) moving in a dynamic environment were selected for the study. The robots are equipped with lidar and IMU sensors. In this section, the kinematic models of the robots and landmarks and the measurement model of the lidar are assumed to be known.

It is worth mentioning that IMUs can provide information on linear acceleration and angular velocity. However, linear speed can be obtained through the integration of linear acceleration.

### 2.1 Kinematic model of the robots

**FIGURE 1:**
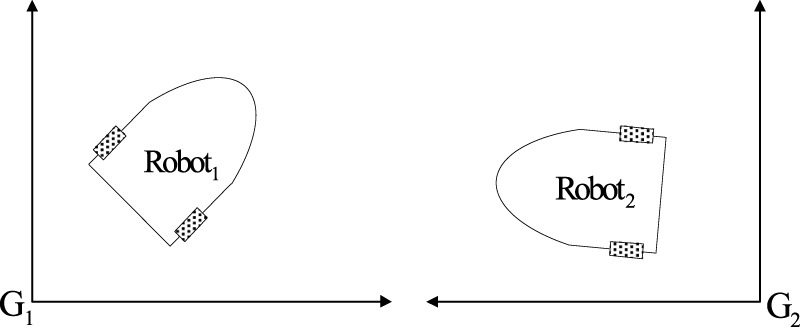
The robots and their corresponding coordinates.

The kinematic model for the two differential drive Pioneer P3-DX mobile robots can be written as follows:

$$\left[\begin{array}{l} x_{r_{i}}\left(k+1\right)\\ y_{r_{i}}\left(k+1\right)\\ \varphi_{r_{i}}\left(k+1\right) \end{array}\right]=\left[\begin{array}{c} x_{r_{i}}\left(k\right)+v\left(k\right)\cos\left(\varphi_{r_{i}}\left(k\right)+\omega \left(k\right)\Delta_{t}\right)\\ y_{r_{i}}\left(k\right)+v\left(k\right)\sin\left(\varphi_{r_{i}}\left(k\right)+\omega \left(k\right)\Delta_{t}\right)\\ \varphi_{r_{i}}\left(k\right)+\omega \left(k\right)\Delta_{t} \end{array}\right]+\upsilon_{r_{i}}\left(k\right),$$ (1)

where $x_{r_{i}}$ and $y_{r_{i}}$ are the *i* ^th^ positions of the robot on the x and y axes and $\varphi_{r_{i}}$ is its corresponding direction relative to the x -axis for i=1,2 . The coordinate system of the first robot is $G_{1}$ and that of the second robot is $G_{2}$ , as depicted in Figure 1. In (1), $\upsilon_{r_{i}}\left(k\right)$ is zero-mean non-Gaussian process noise with a known probability density function (PDF). Additionally, $\Delta_{t}$ is the sampling time of the process and the variables $v\left(k\right)$ and $\omega \left(k\right)$ are the linear and the angular velocities of the robot, which are assumed to be known.

Eq. 1 can be rewritten in the general non-linear state space form as follows:

$$x_{r_{i}}\left(k+1\right)=f_{r_{i}}\left(x_{r_{i}}\left(k\right),u_{r_{i}}\left(k\right)\right)+\upsilon_{r_{i}}\left(k\right),$$ (2)

where $x_{r_{i}}\left(k\right)={\left[\begin{array}{ccc} x_{r_{i}}\left(k\right) & y_{r_{i}}\left(k\right) & \varphi_{r_{i}}\left(k\right) \end{array}\right]}^{T}$ , $u_{r_{i}}\left(k\right)={\left[\begin{array}{cc} \omega \left(k\right) & v\left(k\right) \end{array}\right]}^{T}$ , and $f_{r_{i}}\left(x_{r_{i}}\left(k\right),u_{r_{i}}\left(k\right)\right)$ is the kinematic model presented in (1).

### 2.2 Kinematic model of the landmarks

In this study, both landmarks are considered to be moving, with the following kinematic model:

$$\begin{array}{ll} \left[\begin{array}{l} x_{L_{j},r_{i}}\left(k+1\right)\\ y_{L_{j},r_{i}}\left(k+1\right)\\ \varphi_{L_{j},r_{i}}\left(k+1\right) \end{array}\right] & =\left[\begin{array}{c} x_{L_{j},r_{i}}\left(k\right)+v_{L_{j}}\left(k\right)\cos\left(\varphi_{L_{j},r_{i}}\left(k\right)+\omega_{L_{j}}\left(k\right)\Delta_{t}\right)\\ y_{L_{j},r_{i}}\left(k\right)+v_{L_{j}}\left(k\right)\sin\left(\varphi_{L_{j},r_{i}}\left(k\right)+\omega_{L_{j}}\left(k\right)\Delta_{t}\right)\\ \varphi_{L_{j},r_{i}}\left(k\right)+\omega_{L_{j}}\left(k\right)\Delta_{t} \end{array}\right]\\ & \;+\upsilon_{L_{j},r_{i}}\left(k\right), \end{array}$$ (3)

where $x_{L_{j},r_{i}}$ and $y_{L_{j},r_{i}}$ are the *j* ^th^ positions of the landmark on the x and y axes, respectively, and $\varphi_{L_{j},r_{i}}$ is its corresponding direction with respect to the x -axis for j=1,2 , presented in $G_{i}$ for i=1,2 . Variables $v_{L_{j}}\left(k\right)$ and $\omega_{L_{j}}\left(k\right)$ are the linear velocity and angular velocity of landmark $L_{j}$ , respectively; $\Delta_{t}$ is the sampling time of the process, and k refers to the sample number. It is assumed that $v_{L_{j}}\left(k\right)$ and $\omega_{L_{j}}\left(k\right)$ are known parameters. Moreover, $\upsilon_{L_{j},r_{i}}\left(k\right)$ is a zero-mean Gaussian noise vector: that is, $\upsilon_{L_{j},r_{i}}\left(k\right)∼N\left(0,R_{L_{j},r_{i}}\right)$ , where $N\left(.,.\right)$ refers to the Gaussian PDF.

By writing (3) in the general non-linear state space model, we obtain

$$x_{L_{j},r_{i}}\left(k+1\right)=f_{L_{j},r_{i}}\left(x_{L_{j},r_{i}}\left(k\right),u_{L_{j}}\left(k\right)\right)+\upsilon_{L_{j},r_{i}}\left(k\right),$$ (4)

where $x_{L_{j},r_{i}}\left(k\right)={\left[\begin{array}{ccc} x_{L_{j},r_{i}}\left(k\right) & y_{L_{j},r_{i}}\left(k\right) & \varphi_{L_{j},r_{i}}\left(k\right) \end{array}\right]}^{T}$ , $u_{L_{j}}\left(k\right)={\left[\begin{array}{cc} \omega_{L_{j}}\left(k\right) & v_{L_{j}}\left(k\right) \end{array}\right]}^{T}$ , and $f_{L_{j},r_{i}}\left(x_{L_{j},r_{i}}\left(k\right),u_{L_{j}}\left(k\right)\right)$ is the kinematic model presented in (4).

### 2.3 Lidar measurement model

Lidar sensor output includes the distance and angle of each robot relative to the observed landmarks. Each observation by each robot of the landmarks that are present can be expressed by the following observation model:

$$z_{L_{j},r_{i}}\left(k\right)=\left[\begin{array}{l} r_{L_{j},r_{i}}\left(k\right)\\ \varphi_{L_{j},r_{i}}\left(k\right) \end{array}\right]+\upsilon_{ij},$$

$$r_{L_{j},r_{i}}\left(k\right)=\sqrt{{\left(x_{r_{i}}\left(k\right)-x_{L_{j}}\left(k\right)\right)}^{2}+{\left(y_{r_{i}}\left(k\right)-y_{L_{j}}\left(k\right)\right)}^{2}},$$

$$\varphi_{L_{j},r_{i}}\left(k\right)=\arctan2\left(\frac{y_{r_{i}}\left(k\right)-y_{L_{j}}\left(k\right)}{\left(x_{r_{i}}\left(k\right)-x_{L_{j}}\left(k\right)\right.}\right),$$ (5)

where $z_{L_{j},r_{i}}$ represents the *i* ^th^ robot observation vector of the *j* ^th^ landmark in the environment, $r_{ij}$ represents the distance of the robot from the observed landmark, and $\varphi_{ij}$ is the corresponding angle of the robot relative to the landmark. Additionally, $\upsilon_{ij}$ is a zero-mean Gaussian measurement noise vector with the covariance matrix of $R_{z_{ij}}$ . Moreover, (5) can be rewritten in the general non-linear measurement model as follows:

$$z_{L_{j},r_{i}}\left(k\right)=h\left(x_{r_{i}}\left(k\right),x_{L_{j}}\left(k\right)\right)+\upsilon_{ij}.$$ (6)

## 3 Modified Fast-SLAM with dynamic landmarks

Robots $R_{1}$ and $R_{2}$ explore the environment, with each of them performing the SLAM process from the corresponding frames of $G_{1}$ and $G_{2}$ , respectively; and an independent map of the environment is thereby created by each of them. Information on the distances and angles of the robots relative to the observed landmarks is provided by the lidar sensor in each robot. Each robot then uses a modified Fast-SLAM algorithm. In the proposed modified Fast-SLAM, Rao Blackwellized particle filtering (RBPF) is employed to estimate both vehicle trajectories and landmark positions, in which each landmark is estimated with EKF and PFs are employed to generate particles only used for trajectory estimation. In the remainder of this section, this process is explained, with the application of a modification in EKF due to the presence of moving landmarks with known kinematics. The general steps of Fast-SLAM consist of particle filtering with embedded extended Kalman filtering for the mapping; the data association process; and the map-merging procedure. These steps, with the necessary modifications, are explained for our problem in the following subsections.

### 3.1 Particle generation

An important and initial step in the PF is particle generation. To this end, particles are generated using the known PDF of $\upsilon_{r_{i}}\left(k\right)$ through Monte Carlo simulation, where particles of $\upsilon_{r_{i}}^{l}\left(k-1\right)$ for l=1,...,N are generated and replaced in the motion model of the *i* ^th^ robot (1), and therefore $x_{r_{i}}^{l}\left(k\right)$ , $y_{r_{i}}^{l}\left(k\right)$ , and $\varphi_{r_{i}}^{l}\left(k\right)$ for l=1,...,N and i=1,2 are generated. It is worth mentioning that N refers to the number of particles. This process can be formulated in the following general form according to (2):

$$x_{r_{i}}^{l}\left(k\right)=f_{r_{i}}\left(x_{r_{i}}^{l}\left(k-1\right),u_{r_{i}}\left(k-1\right)\right)+\upsilon_{r_{i}}^{l}\left(k-1\right).$$ (7)

### 3.2 Mapping

Each robot separately performs mapping after the particle generation process using EKF, as explained in the below subsections. After the first meeting of the robots and coordinate transformation, the maps are merged, as explained in detail in subsequent subsections.

#### 3.2.1 EKF for mapping

• Initialization

After the particle generation process as explained in the previous subsection, when the *j* ^th^ landmark is visited for the first time, initialization is performed using the inverse sensor model for each particle, as follows, if it has been visited at sample instant k (neglecting the measurement noise):

$$\begin{array}{l} {\hat{x}}_{L_{j},r_{i}}^{l+}\left(k\right)=x_{r_{i}}^{l}\left(k\right)+r_{L_{j},r_{i}}\left(k\right)\cos\left(\varphi_{L_{j},r_{i}}\left(k\right)+\varphi_{r_{i}}^{l}\left(k\right)\right),\\ {\hat{y}}_{L_{j},r_{i}}^{l+}\left(k\right)=y_{r_{i}}^{l}\left(k\right)+r_{L_{j},r_{i}}\left(k\right)\sin\left(\varphi_{L_{j},r_{i}}\left(k\right)+\varphi_{r_{i}}^{l}\left(k\right)\right),\\ {\hat{\varphi }}_{L_{j},r_{i}}^{l+}\left(k\right)=\varphi_{L_{j},r_{i}}\left(k\right)+\varphi_{r_{i}}^{l}\left(k\right), \end{array}$$ (8)

Where ${\hat{x}}_{L_{j},r_{i}}^{l+}\left(k\right)$ , ${\hat{y}}_{L_{j},r_{i}}^{l+}\left(k\right)$ , and ${\hat{\varphi }}_{L_{j},r_{i}}^{l+}\left(k\right)$ generally refer to the posterior estimates. Although the posterior estimates are obtained in the update step of the Bayesian filtering procedure, as the initial values will be used for prior estimates in the prediction step, a similar notation has been used. (8) can be rewritten in the following general form using (6):

$${\hat{x}}_{L_{j},r_{i}}^{l+}\left(k\right)=h^{-1}\left(z_{L_{j},r_{i}}\left(k\right),x_{r_{i}}^{l}\left(k\right)\right).$$ (9)

Moreover, let predicted covariance matrix $P_{L_{j},r_{i}}^{l+}=KI_{3}$ , where K is a small real number and $I_{3}$ is an identity matrix.

• Prediction step

After the first visit to the *j* ^th^ landmark, prior estimates (predicted estimates) ${\hat{x}}_{L_{j},r_{i}}^{l-}\left(k\right)$ , ${\hat{y}}_{L_{j},r_{i}}^{l-}\left(k\right)$ , and ${\hat{\varphi }}_{L_{j},r_{i}}^{l-}\left(k\right)$ are computed based on the landmark kinematics (3) at each time step for each particle l . Additionally, it is necessary to compute the predicted covariance matrix. This step is the first modification of our method compared with normal Fast-SLAM with static landmarks, in which no kinematics exist for the landmarks. In other words:

$${\hat{x}}_{L_{j},r_{i}}^{l-}\left(k\right)=f_{L_{j},r_{i}}\left({\hat{x}}_{L_{j},r_{i}}^{l+}\left(k-1\right),u_{L_{j}}\left(k-1\right)\right).$$ (10)

$$P_{L_{j},r_{i}}^{l-}\left(k\right)=A_{L_{j},r_{i}}^{l+}P_{L_{j},r_{i}}^{l+}\left(k-1\right){\left(A_{L_{j},r_{i}}^{l+}\right)}^{T}+R_{L_{j},r_{i}},$$ (11)

where in (11) $P_{L_{j},r_{i}}^{l-}$ is the predicted covariance matrix and $P_{L_{j},r_{i}}^{l+}$ is the updated one. Moreover:

$$A_{L_{j},r_{i}}^{l+}={\left.\frac{\partial f_{L_{j},r_{i}}\left(x_{L_{j},r_{i}}\left(k-1\right),u_{L_{j},r_{i}}\left(k-1\right)\right)}{\partial x_{L_{j},r_{i}}\left(k-1\right)}\right|}_{{\hat{x}}_{L_{j},r_{i}}^{l+}\left(k-1\right),u_{L_{j}}\left(k-1\right)}.$$ (12)

• Data association

Data association is one of the important issues for SLAM in dynamic environments. For this purpose, in this paper, the measurement prediction is simply computed as follows:

$$\begin{array}{l} {\hat{z}}_{L_{j},r_{i}}^{i-}\left(k\right)=\left[\begin{array}{l} {\hat{r}}_{L_{j},r_{i}}^{l-}\left(k\right)\\ {\hat{\varphi }}_{L_{j},r_{i}}^{l-}\left(k\right) \end{array}\right],j=1,2\\ {\hat{r}}_{L_{j},r_{i}}^{l-}\left(k\right)=\sqrt{{\left(x_{r_{i}}^{l}\left(k\right)-{\hat{x}}_{L_{j},r_{i}}^{l-}\left(k\right)\right)}^{2}+{\left(y_{r_{i}}^{l}\left(k\right)-{\hat{y}}_{L_{j},r_{i}}^{l-}\left(k\right)\right)}^{2}},\\ {\hat{\varphi }}_{L_{j},r_{i}}^{l-}\left(k\right)=\arctan2\left(\frac{y_{r_{i}}^{l}\left(k\right)-{\hat{y}}_{L_{j},r_{i}}^{l-}\left(k\right)}{\left(x_{r_{i}}^{l}\left(k\right)-{\hat{x}}_{L_{j},r_{i}}^{l-}\left(k\right)\right.}\right), \end{array}$$ (13)

where ${\hat{r}}_{L_{j},r_{i}}^{i-}\left(k\right)$ represents the estimated distance of the *i* ^th^ robot from the *j* ^th^ landmark, and ${\hat{\varphi }}_{L_{j},r_{i}}^{l-}\left(k\right)$ represents the estimated angle of the *i* ^th^ robot relative to *j* ^th^ landmark. Next, the Euclidean distance between the real observations, $z_{L_{j},r_{i}}\left(k\right)$ , and the estimated ones is simply calculated:

$$r_{d_{ij}}^{l}=\left\|z_{L_{j},r_{i}}\left(k\right)-{\hat{z}}_{L_{j},r_{i}}^{l-}\left(k\right)\right\|.$$ (14)

For data association, the condition $r_{d_{ij}}^{l}<\mu$ for j=1,2 is first checked, where μ is a predefined threshold. Subsequently, if this condition is established for both observed landmarks, the smaller value is selected.

• Update step

If the landmark *j* is observed, this step is performed in a similar way to normal Fast-SLAM, which consists of gain computation, state update, and covariance update, as follows:

$$\begin{array}{l} S_{L_{j},r_{i}}^{l}\left(k\right)=H_{L_{j},r_{i}}^{l-}\left(k\right)P_{L_{j},r_{i}}^{l-}\left(k\right){\left(H_{L_{j},r_{i}}^{l-}\left(k\right)\right)}^{T}+R_{z_{ij}},\\ K_{L_{j},r_{i}}^{l}\left(k\right)=P_{L_{j},r_{i}}^{l-}\left(k\right){\left(H_{L_{j},r_{i}}^{l-}\left(k\right)\right)}^{T}{\left(S_{L_{j},r_{i}}^{l}\left(k\right)\right)}^{-1},\\ {\hat{x}}_{L_{j},r_{i}}^{l+}\left(k\right)={\hat{x}}_{L_{j},r_{i}}^{l-}\left(k\right)+K_{L_{j},r_{i}}^{l}\left(k\right)\left(z_{L_{j},r_{i}}\left(k\right)-{\hat{z}}_{L_{j},r_{i}}^{l}\left(k\right)\right),\\ P_{L_{j},r_{i}}^{l+}\left(k\right)=\left(I-K_{L_{j},r_{i}}^{l}\left(k\right)H_{L_{j},r_{i}}^{l-}\left(k\right)\right)P_{L_{j},r_{i}}^{l-}\left(k\right), \end{array}$$ (15)

where $S_{L_{j},r_{i}}^{l}$ is the residual covariance, $K_{L_{j},r_{i}}^{l}$ is the Kalman gain, ${\hat{x}}_{L_{j},r_{i}}^{l+}$ is the updated map of the *j* ^th^ landmark, and $P_{L_{j},r_{i}}^{l+}$ is the updated covariance matrix. Additionally, $H_{L_{j},r_{i}}^{l-}$ is computed as:

$$H_{L_{j},r_{i}}^{l-}={\left.\frac{\partial h\left(x_{r_{i}}\left(k\right),x_{L_{j},r_{i}}\left(k\right)\right)}{\partial x_{L_{j},r_{i}}\left(k-1\right)}\right|}_{{\hat{x}}_{L_{j},r_{i}}^{l-}\left(k\right),x_{r_{i}}^{l}\left(k\right)}.$$ (16)

For unobserved landmarks, the predicted values are replaced with the posterior ones; in other words, $P_{L_{j},r_{i}}^{l+}\left(k\right)=P_{L_{j},r_{i}}^{l-}\left(k\right)$ and ${\hat{x}}_{L_{j},r_{i}}^{l+}\left(k\right)={\hat{x}}_{L_{j},r_{i}}^{l-}\left(k\right)$ .

### 3.3 Localization (weight computation and resampling)

In this step, the final localization process is performed through computation of the particles’ weights and a resampling process. To this end, the normalized weights are computed as follows:

$$w_{L_{j},r_{i}}^{l}\left(k\right)=\frac{p\left(z_{L_{j},r_{i}}\left(k\right)|{\hat{x}}_{L_{j},r_{i}}^{l+}\left(k\right),x_{r_{i}}^{l}\left(k\right)\right)}{\sum_{i=1}^{N}p\left(z_{L_{j},r_{i}}\left(k\right)|{\hat{x}}_{L_{j},r_{i}}^{l+}\left(k\right),x_{r_{i}}^{l}\left(k\right)\right)},$$ (17)

where $w_{L_{j},r_{i}}^{l}\left(k\right)$ is the computed weight for the *l* ^th^ particle corresponding to the *j* ^th^ landmark and the *i* ^th^ robot, and $p\left(z_{L_{j},r_{i}}\left(k\right)|{\hat{x}}_{L_{j},r_{i}}^{l+}\left(k\right),x_{r_{i}}^{l}\left(k\right)\right)$ refers to the conditional PDF. It is worth a reminder that, in this paper, the measurement noise vector is a Gaussian one, and therefore:

$$p\left(z_{L_{j},r_{i}}\left(k\right)|{\hat{x}}_{L_{j},r_{i}}^{l+}\left(k\right),x_{r_{i}}^{l}\left(k\right)\right)=N\left({\hat{z}}_{L_{j},r_{i}}^{l+}\left(k\right),R_{z_{ij}}\right),$$ (18)

where ${\hat{z}}_{L_{j},r_{i}}^{l+}\left(k\right)$ is computed in a similar way to Eq. 13, but replacing ${\hat{x}}_{L_{j},r_{i}}^{l-}\left(k\right)$ and ${\hat{y}}_{L_{j},r_{i}}^{l-}\left(k\right)$ with ${\hat{x}}_{L_{j},r_{i}}^{l+}\left(k\right)$ and ${\hat{y}}_{L_{j},r_{i}}^{l+}\left(k\right)$ , obtained from the update step, respectively. Next, the resampling process is performed using $w_{L_{j},r_{i}}^{l}\left(k\right)$ , and finally a set of N equally weighted particles is generated; these are averaged to produce the final estimate at each sample time k , ${\hat{x}}_{L_{j},r_{i}}\left(k\right)$ and ${\hat{x}}_{r_{i}}\left(k\right)$ .

It is worth mentioning that if no measurement is available, the predicted particles in (7) will be used for particle generation in the next step; additionally, they are weighted equally for the final mapping and localization, ${\hat{x}}_{L_{j},r_{i}}\left(k\right)$ and ${\hat{x}}_{r_{i}}\left(k\right)$ .

## 4 Coordinate alignment and map-merging in multi-robot SLAM

As mentioned earlier, two robots, $R_{1}$ and $R_{2}$ , explore the environment and perform modified Fast-SLAM as explained in the previous section. However, localization and mapping are performed in their corresponding coordinate frames, $G_{1}$ and $G_{2}$ respectively, and each robot is only aware of its own coordinate frame. In order to merge and fuse the separately provided maps, it is necessary for the robots to meet each other. Although there exist many rendezvous approaches to force the robots to meet each other, such as the one presented in (Roy and Dudek, 2001; De Hoog et al., 2010; Zaman et al., 2011; Meghjani and Dudek, 2012; Meghjani and Dudek, 2011), for the sake of simplicity, in this paper it is assumed that the robots travel in such a way that they can meet each other at least once during the mission. When the robots meet each other, it is possible to transform the coordinates. For this purpose, the proposed method of (Zhou and Roumeliotis, 2006) is employed in this paper.

**FIGURE 2:**
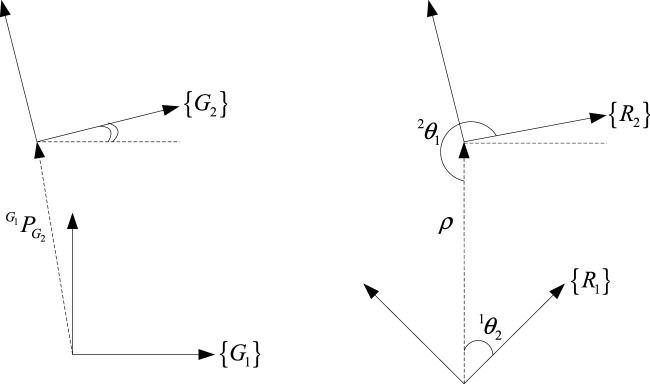
The relationships between different coordinates.

To this end, when robots $R_{1}$ and $R_{2}$ meet using the measurements from the installed lidar sensors, the relative angles between the robots ( θ12 ; θ21 ) and the distance between the robots ( ρ ) are measured, where θ12 is the angle of the first robot as measured by the second one and θ21 is the angle of the second robot measured as by the first. Using the geometrical method presented in (Zhou and Roumeliotis, 2006), the rotation matrix, PG2G1 , and the translation vector, PG2G1 , for transforming a vector in frame $G_{2}$ to frame $G_{1}$ are computed. Readers are referred to (Zhou and Roumeliotis, 2006) for details of the proposed method. Figure 2 clarifies the geometrical relationships between the coordinates and the measurements. Following this, the map created by the second robot can be presented in $G_{1}$ as follows, and the two maps are merged.

x^Lj,r2lkG1=CG2G1x^Lj,r2lk+PG2G1, (19)

where ${\left[{\hat{x}}_{L_{j},r_{2}}^{l}\left(k\right)\right]}_{G_{1}}$ refers to the coordinate of a vector in frame $G_{1}$ .

Finally, all the landmarks are presented in frame $G_{1}$ . If a landmark $L_{j}$ has been visited by both robots, its corresponding map is fused with ${\hat{x}}_{L_{j},r_{1}}^{l}\left(k\right)$ through simple averaging, and it is updated to achieve a more precise map. Moreover, similarly, the trajectory of the second robot can be expressed in $G_{1}$ as a global inertial frame.

Similarly, the map created by the first robot is presented in frame $G_{2}$ using CG1G2 and PG1G2 , where ${\left[{\hat{x}}_{L_{j},r_{1}}^{l}\left(k\right)\right]}_{G_{2}}$ is obtained as follows:

x^Lj,r1lkG2=CG1G2x^Lj,r1lk+PG1G2. (20)

Next, ${\left[{\hat{x}}_{L_{j},r_{1}}^{l}\left(k\right)\right]}_{G_{2}}$ is fused with ${\hat{x}}_{L_{j},r_{2}}^{l}\left(k\right)$ to provide more precise mapping through averaging.

To clarify the proposed method, the general proposed algorithm for multi-robot Fast-SLAM in a dynamic environment is presented and summarized in Algorithm 1.

Algorithm 1 The general multi-robot Fast-SLAM-based algorithm with moving landmarks.

**Step 0: Initialization**
at each sample time k :
 for each robot $r_{i},i=1,2$ :
 for each particle l=1,...,N :
 **Receive**: $x_{r_{i}}^{l}\left(k-1\right)$

  **Step 1: particle generation:** generate particles according to (7).
   **Step 2: Mapping**:
   **Receive**: $P_{L_{j},r_{i}}^{l+}\left(k-1\right)$ , ${\hat{x}}_{L_{j},r_{i}}^{l+}\left(k-1\right)$ .
   for each $L_{j},j=1,2$ :
    • **if** no measurement is available but the landmark has been visited **do:**
     **1.** predict according to (10), (11) and (12)
     **2.** let $P_{L_{j},r_{i}}^{l+}\left(k\right)=P_{L_{j},r_{i}}^{l-}\left(k\right)$ and ${\hat{x}}_{L_{j},r_{i}}^{l+}\left(k\right)={\hat{x}}_{L_{j},r_{i}}^{l-}\left(k\right)$ .
    •**endif**
    • **if** measurement $z_{L_{j},r_{i}}\left(k\right)$ is available **then**:
     • do data association according to (14):
     • **if** the landmark $L_{j}$ is visited for the first time **do**
      **1.** initialization according to (8) and (9).
     • **elseif** the landmark has been visited previously **do**
      **1.** predict according to (10), (11) and (12).
      **2.** update according (15) and (16).
     •**endif**
    •**endif**
  **Step 3: Localization:**
   • **if** measurement $z_{L_{j},r_{i}}\left(k\right)$ is available **do**:
    **1.** generate particle weights according to (17) and (18).
    **2.** perform resampling and generate equally weighted estimates, resulting in:
      
${\left\{\frac{1}{N},{\hat{x}}_{L_{j},r_{i}}^{l+}\left(k\right),x_{r_{i}}^{l}\left(k\right)\right\}}_{l=1}^{N},$

   •**endif**
   • **if** no measurement is available **do**
      
${\left\{\frac{1}{N},{\hat{x}}_{L_{j},r_{i}}^{l+}\left(k\right),x_{r_{i}}^{l}\left(k\right)\right\}}_{l=1}^{N},$

   •**endif**
**  Step 4: Coordinate alignment and map merging:**
   • **if** the robot visits another robot **do**:
    1. compute the rotation matrix, CG2G1 , and the translation vector, PG2G1 .
    2. transform maps in $G_{2}$ to $G_{1}$ according to (19).
    3. transform maps in $G_{1}$ to $G_{2}$ according to (20).
    4. fuse maps as explained in Section 4.
   •**endif**
**Return:** ${\hat{x}}_{L_{j},r_{i}}\left(k\right)$ and ${\hat{x}}_{r_{i}}\left(k\right)$ through averaging.

## 5 Simulation results

To evaluate the performance of the proposed multi-robot SLAM algorithm in a dynamic environment, a series of simulation studies were conducted using MATLAB m-file codes; these are reported on in this section. Two Pioneer P3-DX mobile robots, each equipped with a lidar sensor, are simulated. As mentioned earlier in the article, two moving landmarks are considered and are modeled via their kinematic models.

As mentioned earlier, robots $R_{1}$ and $R_{2}$ explore the environment and observe the landmarks within it; each of them implements the SLAM process from its corresponding frame of $G_{1}$ or $G_{2}$ , respectively, and makes an independent map of the environment. The parameters in our simulation are considered as follows:

$$\begin{array}{l} \omega_{L_{1}}=0.2rad/\sec,\omega_{L_{2}}=0.2rad/\sec,v_{L_{1}}=0.4m/\sec,\\ v_{L_{2}}=0.6m/\sec,\omega =0.2rad/\sec,v=1m/\sec \end{array}$$

All of the sources of noise are considered to be zero-mean Gaussian noise; the covariance matrices of the process (kinematics) noise, for both landmarks and robots, are specified as $0.001I_{3}$ , and the covariance matrices of the lidar noise are specified as $0.001I_{2}$ . Additionally: TG1G2=0.8142−0.5806010.58060.81420−200100001,Δt=0.65⁡sec

$$\begin{array}{l} {\left[x_{R_{1}}\left(0\right)\right]}_{G_{1}}={\left[\begin{array}{ccc} -0.2 & -0.5 & 1.23 \end{array}\right]}^{T},{\left[x_{R_{2}}\left(0\right)\right]}_{G_{1}}={\left[\begin{array}{ccc} 0 & 0 & 2.09 \end{array}\right]}^{T}\\ {\left[x_{L_{1}}\left(0\right)\right]}_{G_{1}}={\left[\begin{array}{ccc} -1.5 & -1 & 1.22 \end{array}\right]}^{T},{\left[x_{L_{2}}\left(0\right)\right]}_{G_{1}}={\left[\begin{array}{ccc} 2.5 & -1.5 & 2.09 \end{array}\right]}^{T} \end{array}$$

Once the robots meet one another, the transformation matrix between the two frames $G_{1}$ and $G_{2}$ is obtained and the maps can be fused to obtain a more precise map of the environment and achieve more precise localization performance. In the case of our simulation, the robots meet each other at 9.35 s and the estimated transformation matrix is as follows:

TG1G2estimated=0.8048−0.593501.09850.59350.80480−1.709800100001

**FIGURE 3:**
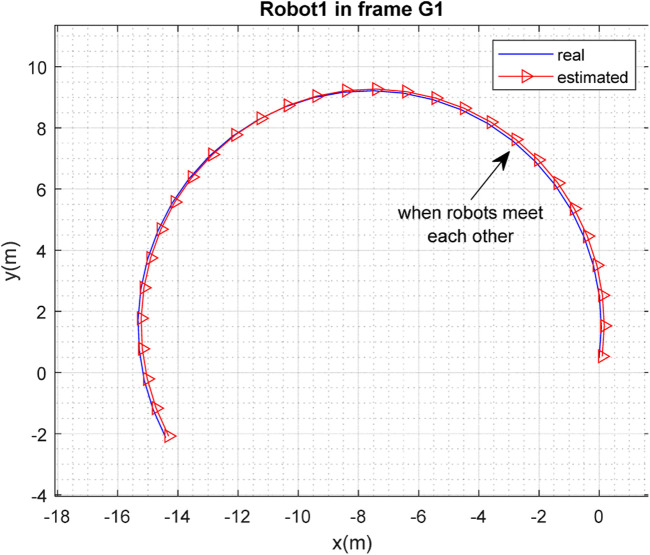
Real and estimated position of robot $R_{1}$ in the frame $G_{1}$ .

**FIGURE 4:**
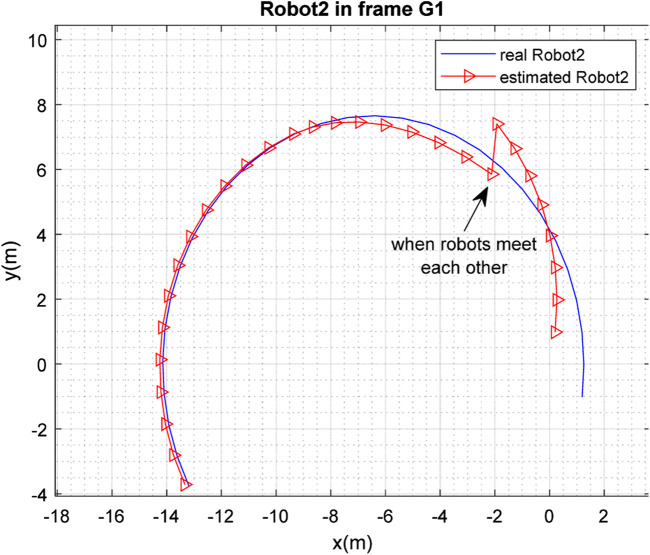
Real and estimated position of robot $R_{2}$ in the frame $G_{1}$ .

**FIGURE 5:**
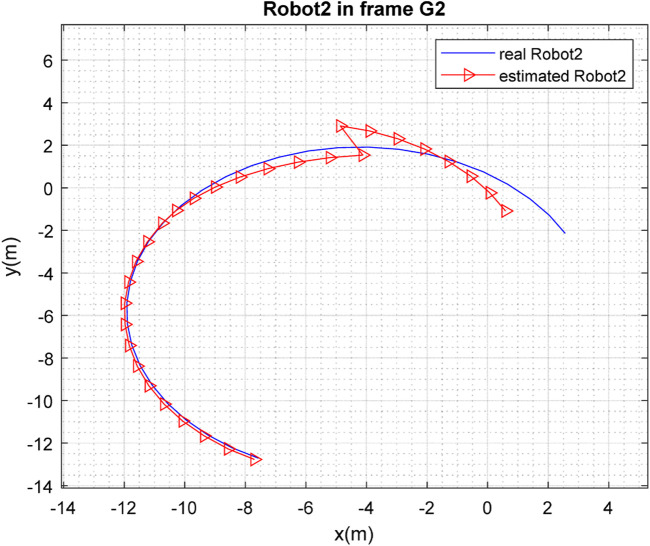
Real and estimated position of robot $R_{2}$ in the frame $G_{2}$ .

Figures 3, 4 depict the results of the localization of robots $R_{1}$ and $R_{2}$ , respectively, in 2D space, expressed in the $G_{1}$ coordinate frame, which is selected as the global frame for this paper. Figure 5 depicts the estimated and real paths of robot $R_{2}$ in frame $G_{2}$ to provide a better illustration of how robot $R_{2}$ is performing localization in its own frame $G_{2}$ .

**FIGURE 6:**
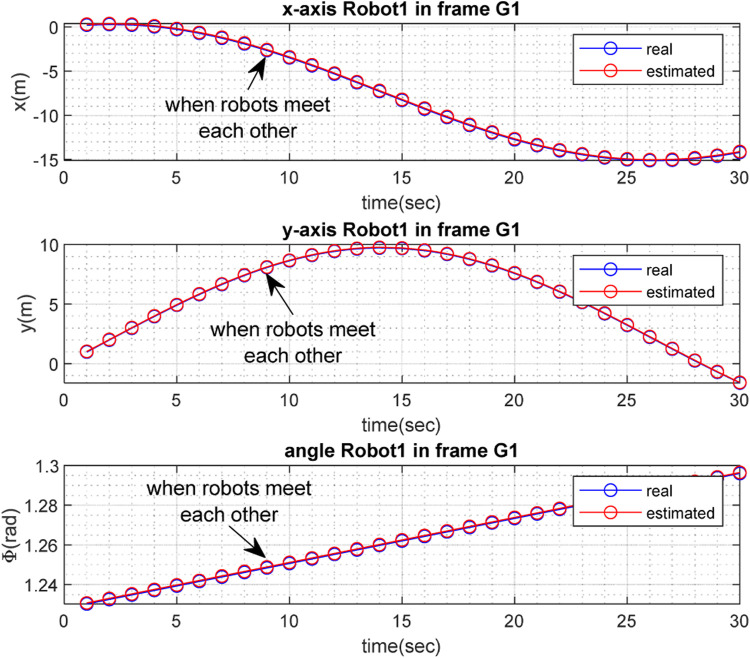
Real and estimated values of $x_{r_{1}}$ , $y_{r_{1}}$ , and $\varphi_{r_{1}}$ in $G_{1}$ .

**FIGURE 7:**
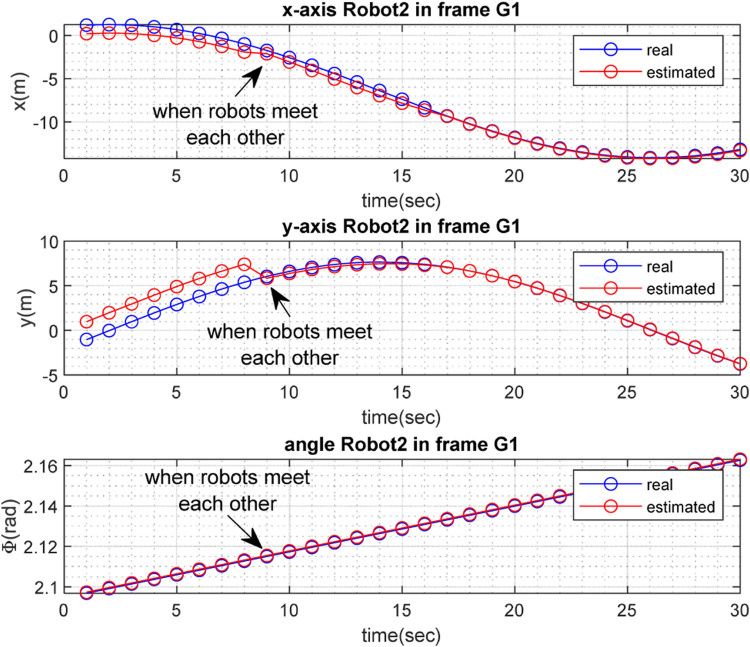
Real and estimated values of $x_{r_{2}}$ , $y_{r_{2}}$ , and $\varphi_{r_{2}}$ in $G_{1}$ .

**FIGURE 8:**
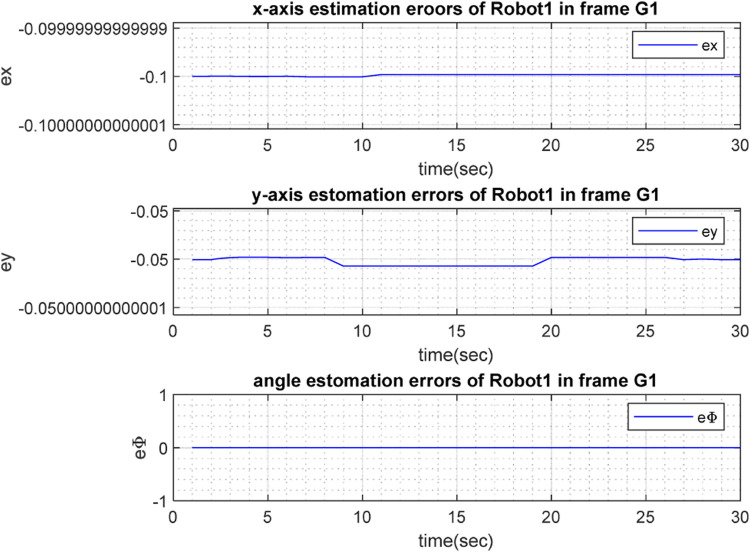
Estimation errors $e_{x_{r_{1}}}$ , $e_{y_{r_{1}}}$ , and $e_{\varphi_{r_{1}}}$ in $G_{1}$ .

**FIGURE 9:**
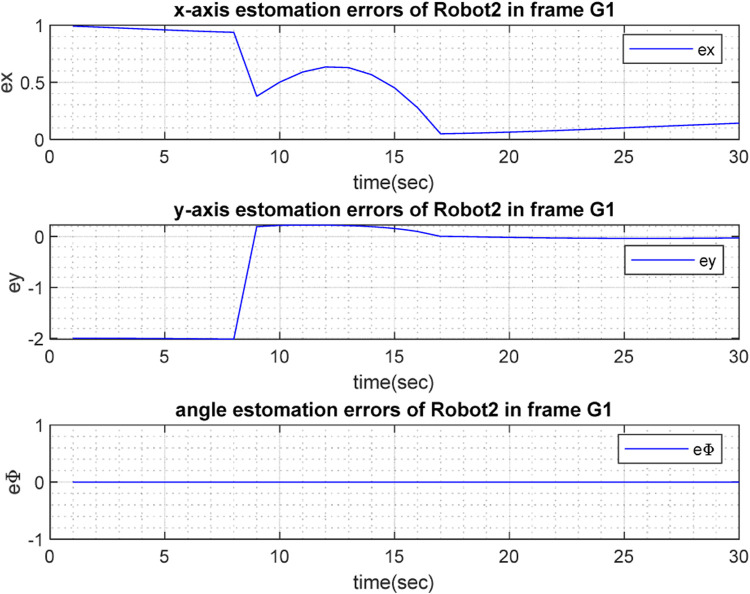
Estimation errors $e_{x_{r_{2}}}$ , $e_{y_{r_{2}}}$ , and $e_{\varphi_{r_{2}}}$ in $G_{1}$ .

The real and estimated values of $x_{r_{i}}$ , $y_{r_{i}}$ , and $\varphi_{r_{i}}$ , expressed in frame $G_{1}$ with respect to time, are depicted in Figures 6, 7 for i=1 (robot $R_{1}$ ) and i=2 (robot $R_{2}$ ), respectively. To show the precision of the estimation, Figures 8, 9 depict the corresponding errors: that is, the error between the real and estimated values ( $e_{x_{r_{i}}}$ , $e_{y_{r_{i}}}$ , and $e_{\varphi_{r_{i}}}$ ) in $G_{1}$ for i=1,2 .

**FIGURE 10:**
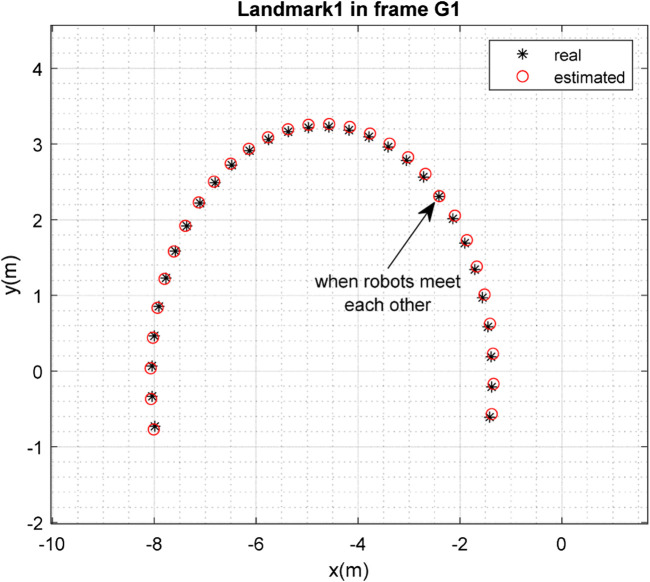
Real position of landmark $L_{1}$ and position estimated by robot $R_{1}$ in the frame $G_{1}$ .

**FIGURE 11:**
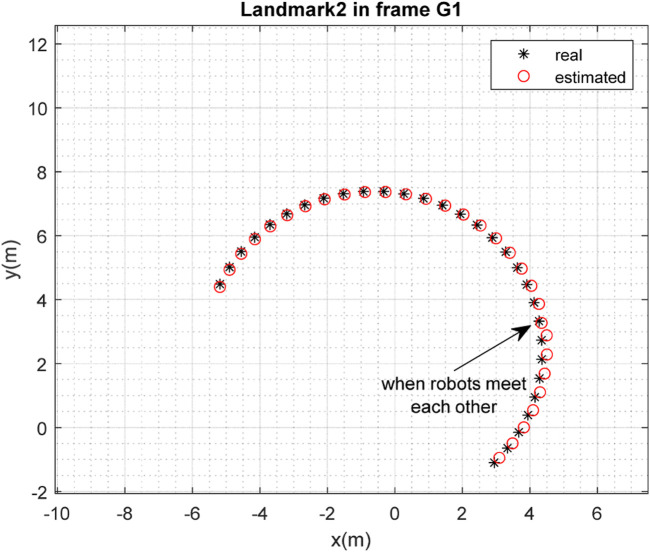
Real position of landmark $L_{2}$ and position estimated by robot $R_{1}$ in the frame $G_{1}$ .

**FIGURE 12:**
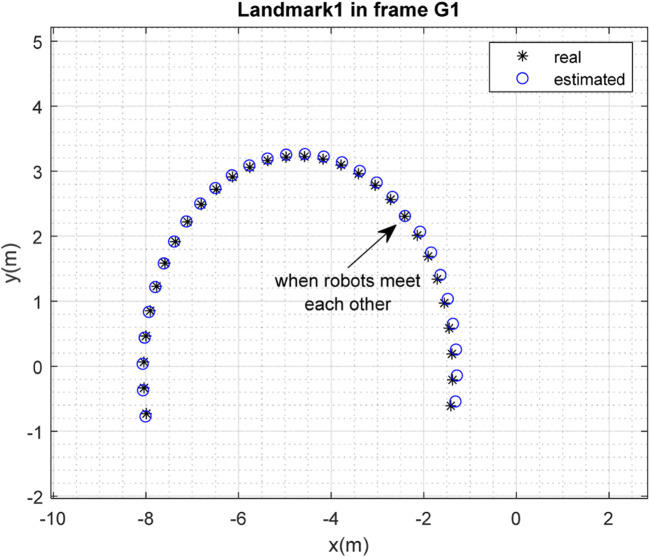
Real position of landmark $L_{1}$ and position estimated by robot $R_{2}$ in the frame $G_{1}$ .

**FIGURE 13:**
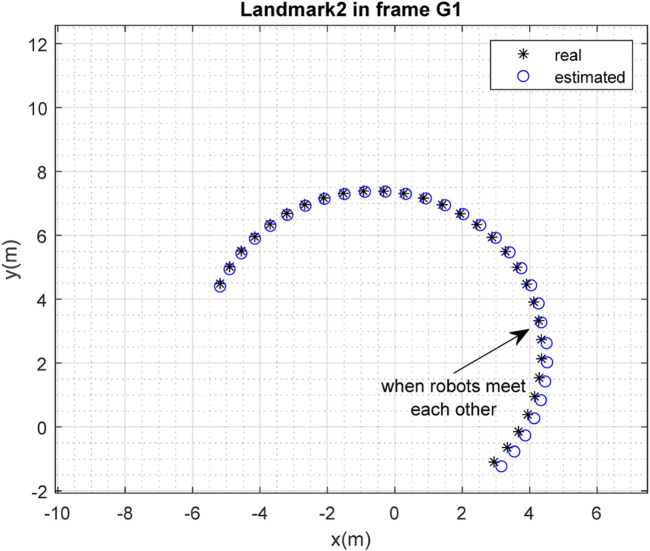
Real position of landmark $L_{2}$ and position estimated by robot $R_{2}$ in the frame $G_{1}$ .

The results of the mapping in 2D space are shown in Figures 10, 11 for landmarks $L_{1}$ and $L_{2}$ obtained from $R_{1}$ , respectively, expressed in frame $G_{1}$ ; the results obtained from $R_{2}$ in frame $G_{1}$ are shown in Figures 12, 13. It is worth mentioning again that when the robots meet each other, the maps obtained from $R_{1}$ and $R_{2}$ are merged. It is clear from the figures that the localization and mapping results are significantly improved after the map-merging process.

To examine the efficiency of the method for the case in which some of the conditions are altered, the initial conditions were selected as follows:

$$\begin{array}{l} {\left[x_{R_{1}}\left(0\right)\right]}_{G_{1}}={\left[\begin{array}{ccc} 0 & 0 & 1.23 \end{array}\right]}^{T},{\left[x_{R_{2}}\left(0\right)\right]}_{G_{1}}={\left[\begin{array}{ccc} 1 & 0 & 2.09 \end{array}\right]}^{T}\\ {\left[x_{L_{1}}\left(0\right)\right]}_{G_{1}}={\left[\begin{array}{ccc} -2.5 & -2.2 & 1.22 \end{array}\right]}^{T},{\left[x_{L_{2}}\left(0\right)\right]}_{G_{1}}={\left[\begin{array}{ccc} 3 & -1 & 2.09 \end{array}\right]}^{T} \end{array}$$

**FIGURE 14:**
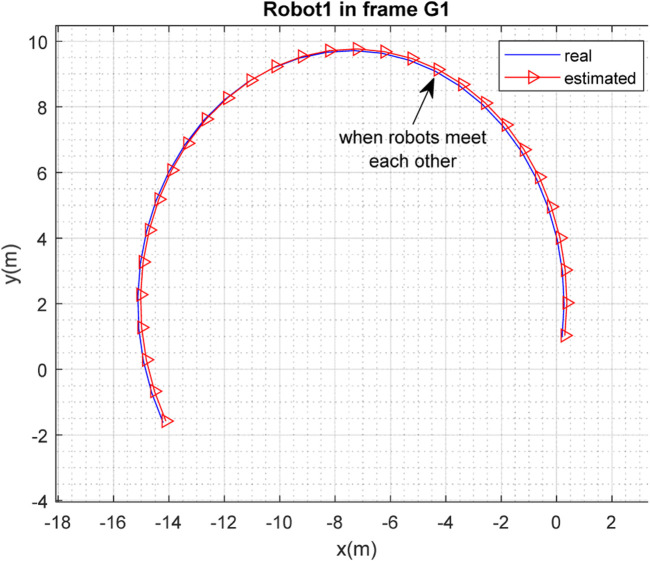
Real and estimated position of robot $R_{1}$ in the frame $G_{1}$ (with different initial conditions).

**FIGURE 15:**
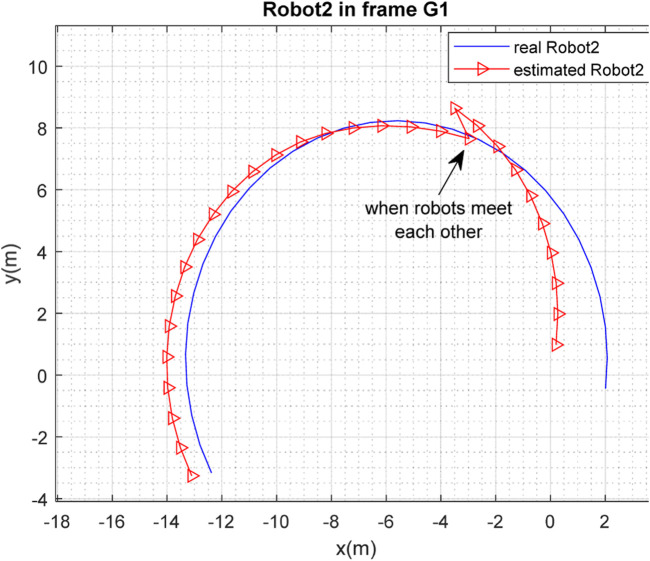
Real and estimated position of robot $R_{2}$ in the frame $G_{1}$ (with different initial conditions).

**FIGURE 16:**
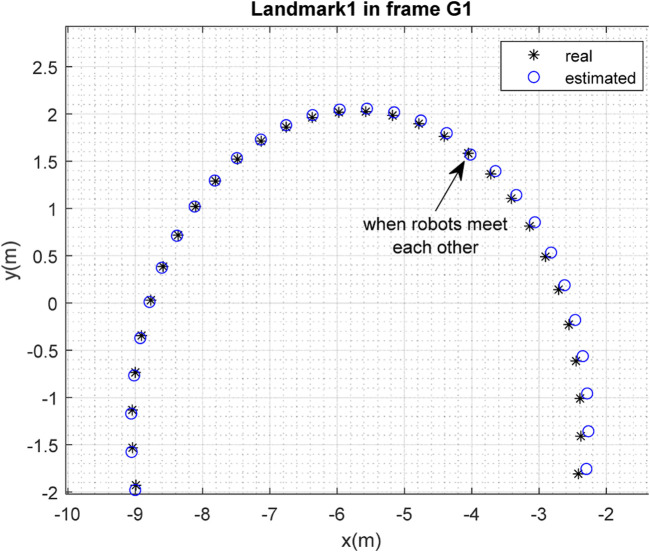
Real position of landmark $L_{1}$ and position estimated by robot $R_{2}$ in the frame $G_{1}$ (with different initial conditions).

**FIGURE 17:**
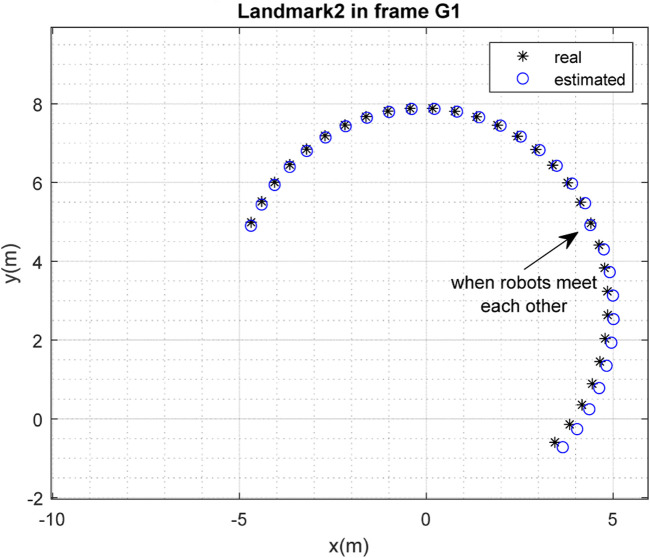
Real position of landmark $L_{2}$ and position estimated by robot $R_{2}$ in the frame $G_{1}$ (with different initial conditions).

**FIGURE 18:**
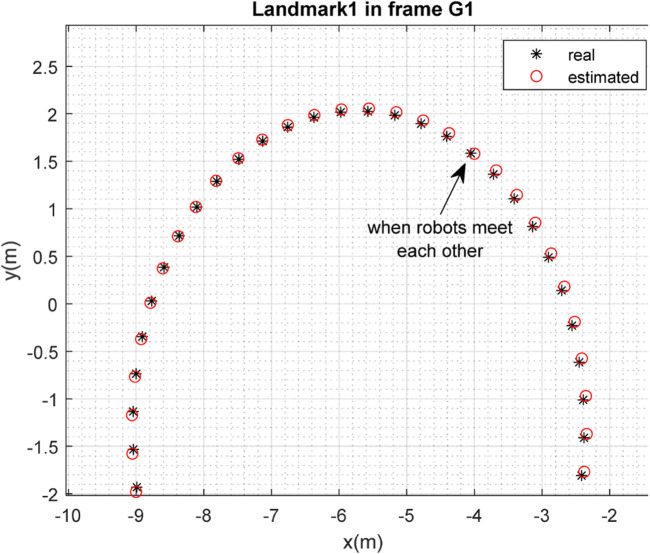
Real position of landmark $L_{1}$ and position estimated by robot $R_{1}$ in the frame $G_{1}$ (with different initial conditions).

**FIGURE 19:**
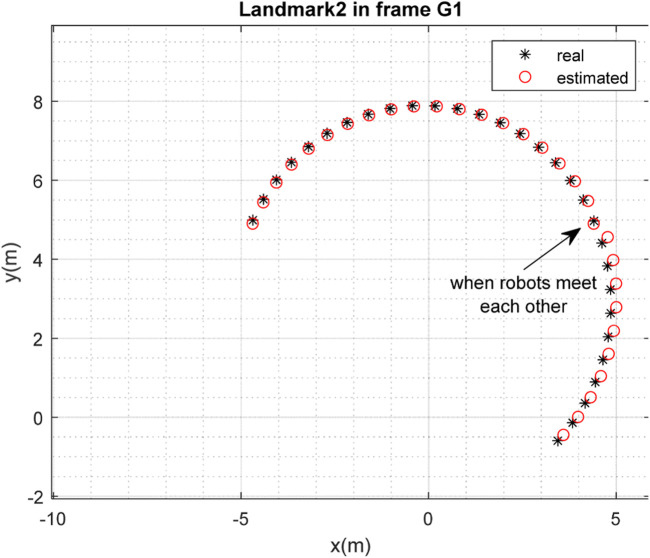
Real position of landmark $L_{2}$ and position estimated by robot $R_{1}$ in the frame $G_{1}$ (with different initial conditions).

The results of localization of the robots in frame $G_{1}$ are depicted in Figures 14, 15, and the results of the mapping are depicted in Figures 16–19. It is clear from the figures that the algorithm is successful in simultaneous localization and mapping under changing initial conditions.

## 6 Conclusion

In this paper, an efficient algorithm has been presented for a multi-robot SLAM problem with unknown initial correspondence in a dynamic environment, using a modified Fast-SLAM method. In our scenario, each robot independently searches the environment, observes the moving landmarks in the environment using a lidar sensor, and implements the SLAM algorithm. In order to distinguish the moving landmarks, kinematic models are considered for the landmarks, which led to a modification of the normal Fast-SLAM method in the form of the addition of a prediction phase to the method; additionally, data association was performed according to the predicted measurements obtained from this prediction step. Although the kinematic models of the landmarks are known within each robot’s coordinate system, after the first meeting of the robots, an initialization is embedded in the algorithm to obtain the current positions of the landmarks, as they are unknown to the robots.

Since the initial correspondence of the robots is unknown (or, in other words, each robot performs the mapping from the perspective of its own coordinate frame), a map-merging procedure was embedded in the proposed algorithm to fuse the independent maps of the robots when the robots meet each other. This map-merging is only possible when the relative transformation matrix of the robots’ inertial frames is computed, which occurs when the robots meet each other. For this purpose, a geographical approach to compute this transformation matrix was embedded in the proposed algorithm.

The performance of the proposed method was evaluated through simulations in MATLAB. It can be concluded from the simulation results that although each robot was able to solve the SLAM problem with an acceptable level of performance, the accuracy of SLAM was significantly improved when the robots met each other and map-merging was performed.

Although the proposed method showed very good performance in simulations in the MATLAB environment, it will perhaps encounter some difficulties in a real environment, such as model mismatches in relation to both the landmarks and the robots due to noise, and the occurrence of drift, for example, in the inertial sensor measurements. Additionally, designing a rendezvous method for the robots to force them to visit each other can represent another issue in a real environment.

Generalization of the proposed method for the general case of a multi-robot and multi-landmark system, with more than two robots and two moving landmarks, is proposed for future work. Embedding some approach to object detection, instead of using the kinematic models of the landmarks, would also add significant value to this research.

## Data Availability

The original contributions presented in the study are included in the article/Supplementary Material, further inquiries can be directed to the corresponding authors.
